# Immunoactive treatment for myasthenia gravis; a Chinese experience

**DOI:** 10.1111/cns.13466

**Published:** 2020-10-26

**Authors:** Nils Erik Gilhus

**Affiliations:** ^1^ Department of Clinical Medicine University of Bergen Bergen Norway; ^2^ Department of Neurology Haukeland University Hospital Bergen Norway

Real‐world long‐term data are important when deciding optimal treatment for a disease. In this issue, Zhang and coworkers report their results from a large multicenter Chinese cohort of patients with chronic, generalized myasthenia gravis (MG).^1^ The outcome for 1064 such patients followed for 6 years at seven independent neurological centers across China shows very good results for rituximab and tacrolimus treatment regarding prevention of MG relapses and drug maintenance.

Placebo‐controlled prospective studies represent the gold standard for new treatments in medicine. Such studies are generally needed before a treatment can be recommended with any strength in guidelines and consensus statements. For neurological disorders such as multiple sclerosis, ischemic stroke, and epilepsy, treatment protocols rely heavily on such studies. This is not the case for MG. There are several reasons for this. MG is a rare disease, and the patients usually do well on present therapy. MG is a fluctuating disease, both during the day and over time. MG has a more limited commercial interest. MG includes several subgroups with differences in pathogenesis and therapeutic response.

Drug effect is demonstrated through placebo‐controlled trials, but therapeutic decisions should ideally be based on controlled studies comparing at least two alternative treatments. Such studies are very rare in MG.^2^ Comparative studies are surprisingly few in medicine in general. A main reason is probably the reluctance of the pharmaceutical industry to undertake such studies and the lack of alternative funding and interest.

In addition to the lack of prospective and controlled studies in MG, it should be acknowledged that such studies do not give all answers. They are usually undertaken only for a limited time period, too short to assess final outcome. The numbers of patients are limited, so that rare side‐effects will not always be acknowledged. The participants are selected regarding age, comorbidity, supplementary treatment, and disease severity.

These limitations illustrate the importance of the present multicenter study from China.^3^ The authors found a MG relapse in only 6% of the patients on rituximab, and 80% of the rituximab‐treated patients continued this drug treatment during the observation period. These results were better than for the other examined treatments. Tacrolimus treatment had also convincing results in this study. The drug was often combined with corticosteroids. This combination had a relapse rate of 13% and drug maintenance similar to rituximab. For therapies with other drugs and drug combinations such as corticosteroids, azathioprine, and mycophenolate mofetil, relapse rates were higher and drug maintenance lower. It is important to remember the limitations of this study. The patients were selected, as all of them should have been in a MG minimal manifestation status or better during the ongoing treatment. More importantly, the various treatments were not given at random to each MG patient but after consideration of which specific treatment was regarded as optimal in each case. This necessarily means selection bias, but on an unknown basis and with unknown consequences for the reported outcome.

Rituximab is increasingly used for MG. In recent guidelines and reviews, this drug is recommended as a primary second‐line drug for moderate and severe generalized MG.^4^, ^5^, ^6^ The effect has most clearly been shown for MG with MuSK antibodies. The only controlled study of rituximab in MG (BeatMG) reported negative results for the primary study outcome, so far only by abstract.^7^ In contrast, several open studies have reported positive results in up to 80% of the patients,^8^, ^9^ similar to Zhang et al^1^ Retention rates for rituximab in MG have not been communicated until this new Chinese study. However, in MS, rituximab is clearly superior to all other examined treatments regarding maintenance over time. In a Swedish study, the annual discontinuation rate was only 3% for rituximab, compared with 30%‐50% for the other MS drugs examined.^10^ This reflects a very good tolerability in addition to the consistent immunosuppressive effect. PML is the most feared side effect of rituximab. It is rare, occurring in less than 1:30 000 treated patients.^11^ Monotherapy, such as in most of the Chinese patients, further reduces the PML risk. From pathogenic considerations as well, one would expect rituximab to be a favored treatment for an antibody‐mediated disease such as MG. The drug binds selectively to CD20, an antigen primarily found on the cell membrane of the B‐lymphocyte line.

Tacrolimus has similarities to cyclosporine. Controlled trials assessing the steroid‐sparing effect of tacrolimus in MG gave conflicting results, but tended to show a positive effect.^12^, ^13^ Tacrolimus has in addition to immunosuppression an effect on ryanodine receptor‐mediated calcium release from the muscle cell sarcoplasmic reticulum, which theoretically might be beneficial in MG. The drug seems to be more widely used for MG in the Far East than in Europe and North America. 633 MG patients treated with tacrolimus in 24 different studies were recently pooled in a meta‐analysis.^2^ The authors concluded with a significant positive drug effect and mostly mild side‐effects. The present article from China further supports the use of tacrolimus in combination with corticosteroids as an effective and well‐tolerated treatment in generalized MG.

Treatment of MG should consider a series of therapeutic elements and always be adapted to the individual patient. Early thymectomy should be undertaken in early‐onset generalized MG with AChR antibodies, in selected patients with late‐onset MG, in nearly all patients with thymoma, and even in some patients with ocular MG.^4^ More than 90% of patients with active MG use pyridostigmine as symptomatic treatment. Physical training programmes improve both muscle strength and general health in MG, well documented in controlled trials. Comorbidity represents a challenge for precise diagnosis of weakness, reduced health, and exacerbations in elderly MG patients, and also for optimal treatment.^14^ Special situations such as pregnancy, surgery, and epidemics require individualized treatment considerations.

MG treatment is based on published controlled studies, uncontrolled studies, consensus reports, and recommendations from experts, as well as local experience and tradition. The multicenter data from China reported in this issue^1^ add new and important information regarding such treatment. The large number of MG patients and the long follow‐up time give weight to their conclusions. The size of the patient material and the applied methods make comparisons between different immunosuppressive treatment regimens meaningful, although unaccounted bias is an obvious weakness in an uncontrolled study. Their recommendation of more extensive use of rituximab is in line with reports and experience from many centers worldwide. Their recommendation of tacrolimus as an effective and safe drug should be considered. More data regarding tacrolimus treatment of MG also outside the Far East would be of high interest. Multicenter data sets are especially welcome when evaluating best treatment for rare diseases. They make it possible to include a sufficiently high number of patients to compare various treatments. The present Chinese article shows how such studies could be performed.
